# High grade non-Hodgkin lymphoma as culprit of ileal perforation in a patient presenting with generalized peritonitis: a case report

**DOI:** 10.1186/s13256-025-05819-6

**Published:** 2026-01-20

**Authors:** Wondwosen Mengist Dereje, Asratu Getnet Amare, Cheru Lilay Gebrehiwet, Eyoel Negash Taddesse, Endalew Demoz Worku, Hailemariam Yohannes Asefa, Emebet Hunie Bazie, Endeshaw Asaye Kindie

**Affiliations:** 1 Department of Neurology, University of Gondar College of Medicine and Health, Gondar, Ethiopia; 2 Department of Surgery, University of Gondar College of Medicine and Health, Gondar, Ethiopia; 3 Department of Clinical Oncology, University of Gondar College of Medicine and Health, Gondar, Ethiopia; 4 Department of Pathology, University of Gondar College of Medicine and Health, Gondar, Ethiopia

**Keywords:** Lymphoma, Anaplastic type, Perforation, Laparotomy, Chemotherapy, Case report

## Abstract

**Background:**

Non-Hodgkin lymphoma is one of the most common types of lymphoma affecting the gastrointestinal tract. This malignancy can originate from either B-lymphocytes or T-lymphocytes. Intestinal perforation due to non-Hodgkin lymphoma is a relatively rare occurrence, and when it does happen, it typically arises after the initiation of chemotherapy. In the reported case, the perforation happened prior to the chemotherapy, making the case unusual. We aim to highlight the importance of maintaining a high index of suspicion and the critical role of timely, multidisciplinary management in improving patient outcomes.

**Clinical presentation:**

A 27-year-old male farmer from a rural area in North West Gondar, Ethiopia, was referred to our hospital for further evaluation and management. He initially presented to the referring facility with a 1-week history of abdominal pain. The pain began as a periumbilical discomfort during the first 5 days and later progressed to become diffuse, involving all regions of the abdomen. He also reported three episodes of vomiting and a high-grade fever that had persisted for the last 2 days. Due to lack of timely histopathologic evaluation, confirmatory diagnosis was done after 1 month of surgical intervention.

**Conclusion:**

Intestinal perforation caused by lymphoma is a rare occurrence in clinical practice, especially before the initiation of chemotherapy. When such perforation does occur, it is often misdiagnosed or diagnosed late due to its atypical clinical presentation. In many cases, the diagnosis is only established postoperatively following histopathological examination.

## Background

Non-Hodgkin lymphoma (NHL) is the most common form of gastrointestinal (GI) lymphoma and is associated with several serious complications, including bleeding, obstruction, and perforation [1].

NHL of the ileum is a type of intestinal tumor that arises from intraepithelial T-lymphocytes. It typically presents as a neoplasm composed of large lymphoid cells and is often accompanied by necrosis, an inflammatory background, and a notable presence of histiocytes and eosinophils [2].

The gastrointestinal (GI) tract represents the most frequent extranodal site of involvement in lymphoma cases [3].

## Case presentation

A 27-year-old male farmer from rural Northwest Gondar, Ethiopia, was referred to our tertiary hospital with a diagnosis of generalized peritonitis secondary to a suspected perforated viscus. The referral was made due to the unavailability of a surgeon, essential staff, and surgical materials at the primary hospital.

Initially, the patient presented to the primary hospital with a 1-week history of dull, aching, nonradiating abdominal pain. During the first 5 days, the pain was localized to the periumbilical region. However, 2 days before referral, the pain became diffuse, involving all quadrants of the abdomen. This change was accompanied by a loss of appetite. Subsequently, he developed three episodes of nonprojectile, nonbilious vomiting of ingested food, along with a high-grade fever.

At the primary hospital, initial management included intravenous resuscitation with 2 L of normal saline and administration of ceftriaxone 1 g intravenous and metronidazole 500 mg intravenous. Due to lack of surgical services, the patient was referred to our facility for further evaluation and management.

Upon arrival at our hospital, the patient was in severe pain. His vital signs were as follows: blood pressure 110/70 mmHg, pulse rate 120 beats per minute, respiratory rate 20 breaths per minute, and a temperature of 38.9 °C. Anthropometric evaluation revealed body mass index (BMI) of 23.7 kg/m^2^. Abdominal examination showed diffuse tenderness across all quadrants, consistent with peritonitis. All other systemic examinations were normal.

Two large-bore intravenous lines were secured, and the patient was started on intravenous fluids (normal saline) and antibiotics (ceftriaxone 1 g intravenous and metronidazole 500 mg intravenous). A Foley catheter was inserted for urine output monitoring, and blood samples were collected for laboratory investigations and blood typing. With a working diagnosis of generalized peritonitis secondary to a perforated viscus—most likely perforated appendicitis—the patient was immediately taken to the operating room for an emergency exploratory laparotomy.

Preoperative laboratory investigations showed:

- Complete blood count (CBC): WBC 23.4 × 10^3^/µL (80% neutrophils, 12% lymphocytes), hemoglobin 14.5 g/dL, hematocrit 47.3%, platelet count 187 × 10^3^/µL
- Renal function: serum creatinine 0.76 mg/dL
- Electrolytes: Na^+^ 143 mmol/L, K^+^ 4.5 mmol/L, Cl^−^ 113 mmol/L, Ca^2+^ 1.27 mmol/L
- Liver enzymes: SGOT 66 U/L, SGPT 45 U/L
- Blood culture: No growth

**Fig. 1:**
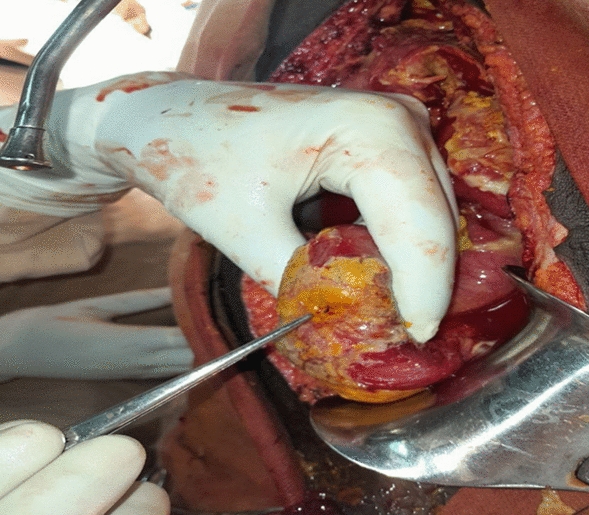
The perforated ileal segment on the antimesenteric side (intraoperative image)

**Fig. 2:**
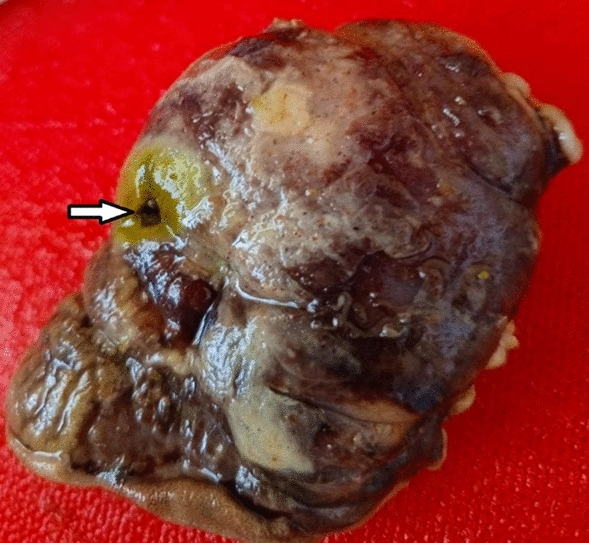
The resected segment of the bowel sent for histopathologic evaluation with visible full layer perforation on the ileum (arrow)

Intraoperative exploration revealed a 1 × 1.5 cm full-thickness perforation on the antimesenteric side of the ileum (Fig. 1). Approximately 1500 mL of purulent peritoneal fluid mixed with intestinal contents was drained. The affected segment of the ileum was resected, and primary anastomosis was performed. A specimen from the resected segment was sent for histopathological examination (Fig. 2).

Due to limited pathology resources, a high sample load, and a shortage of pathologists, the patient was given an appointment to return in 1 month for his histopathology results. Postoperatively, the patient had an uneventful recovery. He was closely monitored for 4 days without any complications and was discharged with scheduled follow-up at the surgical outpatient clinic.

**Fig. 3:**
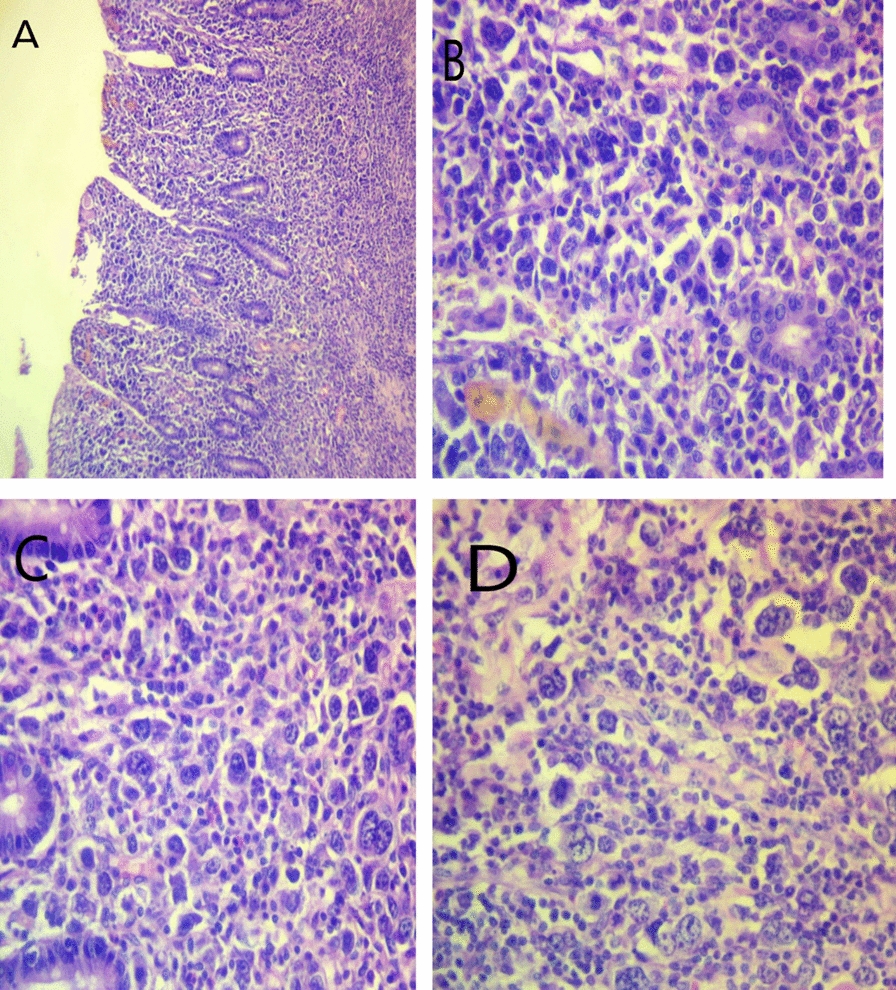
Histopathologic images after hematoxylin and eosin staining highly suggestive of anaplastic large cell lymphoma (ALCL). **A** Lymphoid cells invading the mucosa with crypt destruction and scattered hallmark lymphoid cells. **B** Monotonous proliferations of medium-to-large sized lymphoid cells having frequent mitotic activities. **C** Monotonous proliferations lymphoid cells with crypt destructions. **D** Lymphoid cells with irregular hyperchromatic nuclei

One month later, the patient returned with the histopathology result, which revealed high-grade non-Hodgkin lymphoma (NHL), suggestive of anaplastic large cell lymphoma (ALCL); lymphoid cells invading the mucosa (Fig. 3A), lymphoid cells with frequent mitotic activities (Fig. 3B), crypt destruction (Fig. 3C), and irregular hyperchromatic nuclei (Fig. 3D).

This diagnosis was discussed in detail with the patient and his family. Following oncologic consultation and evaluation, he was admitted to the oncology unit, where chemotherapy with cyclophosphamide, doxorubicin, vincristine, and prednisolone (CHOP) regimen was initiated. He is motivated and happy with the help he is getting from his families, health professionals, and everyone around him.

## Discussion

Primary lymphomas of the gastrointestinal (GI) tract are relatively rare. While the stomach is the most commonly affected site, any part of the digestive system—including the esophagus and rectum—can be involved. In contrast, secondary gastrointestinal involvement is more frequently observed due to the widespread nature of nodal disease. The majority of GI lymphomas are classified as non-Hodgkin lymphomas [4].

Previous studies have indicated that primary intestinal lymphoma predominantly affects male individuals, with a reported male-to-female ratio of approximately 2.5:1. The clinical presentation of small intestinal lymphoma is generally nonspecific. Most patients present with acute abdominal pain (70–80%), while other common symptoms include weight loss (30%), hematochezia (25.9%), diarrhea (16.9%), nausea, vomiting, and loss of appetite [5–7]. However, complications such as intestinal perforation and acute bowel obstruction are relatively rare [8].

The nonspecific clinical manifestations of non-Hodgkin lymphoma (NHL) make preoperative diagnosis particularly challenging. In most cases, a definitive diagnosis can only be established through postoperative histopathological examination.

Due to its rarity and low incidence, intestinal perforation caused by lymphoma is frequently overlooked and often misdiagnosed as other gastrointestinal conditions such as Crohn’s disease, intestinal tuberculosis, or typhoid enteritis. In patients presenting with abdominal pain, fever, gastrointestinal perforation, and hematochezia—especially when multiple intestinal wall ulcers are detected during colonoscopy—the possibility of lymphoma should be considered. This awareness is crucial to avoid misdiagnosis or delayed diagnosis [9].

Intestinal perforation is a well-recognized complication of non-Hodgkin lymphoma (NHL) and may occur either at the time of initial presentation or following the initiation of chemotherapy. Predicting the risk of perforation before treatment remains difficult, and currently, there is no established method to reliably prevent such serious gastrointestinal complications in these patients [10].

In individuals undergoing chemotherapy, the causes of perforation include rapid tumor necrosis, tumor lysis, and tissue damage resulting from excessive granulation [11]. The incidence of intestinal perforation following chemotherapy in patients with intestinal lymphoma is approximately 9%. Among gastrointestinal lymphoma cases, the small intestine is the most frequently affected site, with diffuse large B-cell lymphoma (DLBCL) being the most common subtype associated with perforations [12].

Although the exact incidence of intestinal perforation prior to chemotherapy is not well documented in the literature, it is considered rare. This rarity is likely due to the fact that tumor necrosis and tissue damage, which are key contributors to perforation, typically occur following the initiation of chemotherapy.

In our case, the patient developed ileal perforation prior to the initiation of chemotherapy, making this presentation particularly uncommon and clinically noteworthy.

Slightly more than half of gastrointestinal perforations following chemotherapy occur within the first month or during the initial treatment cycle, while the remaining cases present more than 4 weeks after chemotherapy has begun. These perforations can result not only from treatment-related complications such as neutropenic colitis, infectious colitis, radiation enteritis, and colonic pseudo-obstruction, but also from the extensive infiltration of the gastrointestinal tract by lymphoma itself. Certain preoperative factors, including smoking, comorbid conditions such as diabetes mellitus, elevated lactate dehydrogenase (LDH) levels, hypoalbuminemia, and the presence of T-cell lymphoma, are considered independent risk factors for perforation in patients with gastrointestinal lymphoma [13].

Other case series have reported a wide range in the timing of perforation, from as early as 4 days to more than 5 weeks after initiating chemotherapy. Additionally, the use of corticosteroids may mask clinical symptoms, allowing a perforation to occur without being immediately detected [14].

Gastrointestinal (GI) perforation is a potentially life-threatening condition, particularly in patients who are already debilitated and immunocompromised following chemotherapy. There is ongoing debate regarding the optimal surgical approach for managing GI perforation in these cases. Primary resection with stoma formation is generally preferred, especially in the presence of generalized peritonitis or when dealing with large, unresectable tumors. Primary resection with anastomosis is typically avoided due to the heightened risk of anastomotic leakage in immunosuppressed patients. However, emerging evidence suggests that, under select surgical conditions and with advanced techniques, resection followed by primary anastomosis may be performed safely [15].

In our case, the patient underwent primary resection with anastomosis, as lymphoma was not initially suspected until the histopathological diagnosis was made postoperatively. Notably, the patient did not experience any postoperative complications.

In general, the recommended treatment approach for non-Hodgkin lymphoma (NHL) involving the gastrointestinal tract is surgical intervention followed by chemotherapy. Specifically, surgery followed by adjuvant multiagent chemotherapy, typically a combination of cyclophosphamide, doxorubicin, vincristine, and prednisolone (CHOP), has been associated with improved outcomes. In most cases, NHL is managed with anthracycline-based regimens, with CHOP being the standard of care [16].

Despite advances in treatment, the overall prognosis for patients with NHL remains poor [17, 18]. Reported 5-year survival rates are highly variable, ranging from as low as 8% to as high as 60%, reflecting the heterogeneity of the disease and influencing factors such as histologic subtype, disease stage, and patient condition [19, 20].

Due to lack of immunohistochemistry in our setting and economical problems of the patient to send the sample to the capital city for evaluation, the morphologic appearances of tissue is highly suggestive of ALCL. After thorough communication with the clinical oncologist, the patient was started on CHOP regimen chemotherapy, and 3 months after the initiation of his chemotherapy regimen, he is getting stronger and motivated at each visit to our unit. He is optimistic about the future and enthusiastic about the effect he saw from the treatment.

Our patient is a young and motivated male individual with no comorbidities. Although the likely histopathologic type is ALCL, considering the above conditions, we are hopeful that he has a good chance of a better survival rate.

## Conclusion

Intestinal perforation secondary to non-Hodgkin lymphoma (NHL) is a rare but potentially life-threatening condition, especially when it occurs before the initiation of chemotherapy. Its atypical clinical presentation often leads to delayed or missed diagnosis, with many cases only confirmed postoperatively through histopathological evaluation. Such delays in diagnosis and treatment initiation can result in poor clinical outcomes. Therefore, maintaining a high index of suspicion is essential, particularly in patients presenting with acute abdomen of unclear origin. Early diagnosis, timely intervention, and a multidisciplinary approach are critical to improving patient prognosis and reducing the risk of complications.

## Data Availability

Due to privacy of the patient we decline to share data.

## Abbreviations

NHL: Non-Hodgkin lymphoma
SGOT: Serum glutamic-oxaloacetic transaminase
SGPT: Serum glutamic-pyruvic transaminase
CBC: Complete blood count
WBC: White blood cell
ALCL: Anaplastic large cell lymphoma
DLBCL: Diffuse large B-cell lymphoma
LDH: Lactate dehydrogenase
CHOP: Cyclophosphamide, doxorubicin, vincristine, and prednisolone

## References

[1] Ponnusamy R, Dasgupta P, Pai A. Intestinal perforation in a case of peripheral T cell lymphoma after initiation of chemotherapy. Korean J Gastroenterol. 2024;84(2):90–4. 10.4166/kjg.2024.072. 39176464 10.4166/kjg.2024.072 PMC12285490

[2] Ferreri AJM, Zinzani PL, Govi S. Enteropathy-associated T-cell lymphoma. Crit Rev Oncol Hematol. 2011;79:84–90. 10.1016/j.critrevonc.2010.06.006. 20655757 10.1016/j.critrevonc.2010.06.006

[3] Gou HF, Zang J, Jiang M, Yang Y, Cao D, Chen XC. Clinical prognostic analysis of 116 patients with primary intestinal non-Hodgkin lymphoma. Med Oncol. 2012;29:227–34. 21193968 10.1007/s12032-010-9783-x

[4] Ghimire P, Wu GY, Zhu L. Primary gastrointestinal lymphoma. World J Gastroenterol. 2011;17:697–707. 21390139 10.3748/wjg.v17.i6.697 PMC3042647

[5] Ara C, Coban S, Kayaalp C, Yilmaz S, Kirimlioglu V. Spontaneous intestinal perforation due to non-Hodgkin’s lymphoma: evaluation of eight cases. Dig Dis Sci. 2007;52(8):1752–6. 10.1007/s10620-006-9279-x. 17420936 10.1007/s10620-006-9279-x

[6] Ding D, Pei W, Chen W, Zuo Y, Ren S. Analysis of clinical characteristics, diagnosis, treatment and prognosis of 46 patients with primary gastrointestinal non-Hodgkin lymphoma. Mol Clin Oncol. 2014;2(2):259–64. 10.3892/mco.2013.224. 24649343 10.3892/mco.2013.224 PMC3917777

[7] Wang GB, Xu GL, Luo GY, Shan HB, Li Y, Gao XY, Li JJ, Zhang R. Primary intestinal non-Hodgkin’s lymphoma: a clinicopathologic analysis of 81 patients. World J Gastroenterol. 2011;17(41):4625–31. 10.3748/wjg.v17.i41.4625. 22147970 10.3748/wjg.v17.i41.4625 PMC3226984

[8] Li B, Shi YK, He XH, Zou SM, Zhou SY, Dong M, Yang JL, Liu P, Xue LY. Primary non-Hodgkin lymphomas in the small and large intestine: clinicopathological characteristics and management of 40 patients. Int J Hematol. 2008;87(4):375–81. 10.1007/s12185-008-0068-5. 18409078 10.1007/s12185-008-0068-5

[9] Giuliani A, Romano L, Coletti G, Walid A Fatayer M, Calvisi G, Maffione F, Muolo C, Vicentini V, Schietroma M, Carlei F. Lymphangiomatosis of the ileum with perforation: a case report and review of the literature. Ann Med Surg. 2019;41:6–10. 10.1016/j.amsu.2019.03.010. 10.1016/j.amsu.2019.03.010 PMC6449703 30992989

[10] Imataki O, Shiroshita K, Uchida S, Kida J, Akamoto S, Uemura M. Perforation in an intestinal malignant lymphoma case. BMC Res Notes. 2016;9(1): 308. 10.1186/s13104-016-2111-6. 27297406 10.1186/s13104-016-2111-6 PMC4906586

[11] Tatar C, Yavas M, Akkus O, Tapkan B, Batikan OK, Bayrak S, Arikan S. Intestinal perforation that developed after chemotherapy in a patient diagnosed with non-Hodgkin lymphoma: a case report and review of literature. Int J Surg Case Rep. 2017;39:321–3. 10.1016/j.ijscr.2017.08.058. 28898795 10.1016/j.ijscr.2017.08.058 PMC5597875

[12] Vaidya R, Witzig TE. Incidence of bowel perforation in gastrointestinal lymphomas by location and histology. Ann Oncol. 2014;25(6):1249–50. 10.1093/annonc/mdu135. 24692578 10.1093/annonc/mdu135 PMC4110448

[13] Vaidya R, Habermann TM, Donohue JH, Ristow KM, Maurer MJ, Macon WR, Colgan JP, Inwards DJ, Ansell SM, Porrata LF, Micallef IN, Johnston PB, Markovic SN, Thompson CA, Nowakowski GS, Witzig TE. Bowel perforation in intestinal lymphoma: incidence and clinical features. Ann Oncol. 2013;24(9):2439–43. 10.1093/annonc/mdt188. 23704194 10.1093/annonc/mdt188 PMC3755328

[14] Abbott S, Nikolousis E, Badger I. Intestinal lymphoma--A review of the management of emergency presentations to the general surgeon. Int J Colorectal Dis. 2015;30(2):151–7. 10.1007/s00384-014-2061-1. 25374417 10.1007/s00384-014-2061-1

[15] Ahmed G, ElShafiey M, Abdelrahman H, Semary S, Elkinaai N, Romeih M, Mohy R, Younes A. Surgery in perforated pediatric intestinal lymphoma. Eur J Surg Oncol. 2019;45(2):279–83. 10.1016/j.ejso.2018.08.022. 30224248 10.1016/j.ejso.2018.08.022

[16] Sun ZH, Zhou HM, Song GX, Zhou ZX, Bai L. Intestinal T-cell lymphomas: a retrospective analysis of 68 cases in China. World J Gastroenterol. 2014;20(1):296–302. 10.3748/wjg.v20.i1.296. 24415885 10.3748/wjg.v20.i1.296 PMC3886022

[17] Tang XF, Yang L, Duan S, Guo H, Guo QN. Intestinal T-cell and NK/T-cell lymphomas: a clinicopathological study of 27 Chinese patients. Ann Diagn Pathol. 2018;37:107–17. 10.1016/j.anndiagpath.2018.10.004. 30317149 10.1016/j.anndiagpath.2018.10.004

[18] Ma WL, Yeh KH, Yao M, Tang JL, Lin CW, Wang YT, Yeh YC, Wang HP, Cheng AL, Kuo SH. Comparison of clinicopathological features and treatment outcomes in aggressive primary intestinal B-and T/NK-cell lymphomas. J Formos Med Assoc. 2021;120(1 Pt 2):293–302. 10.1016/j.jfma.2020.10.001. 33289640 10.1016/j.jfma.2020.10.001

[19] Spijkerman M, Tan IL, Kolkman JJ, Withoff S, Wijmenga C, Visschedijk MC, Weersma RK. A large variety of clinical features and concomitant disorders in celiac disease - A cohort study in the Netherlands. Dig Liver Dis. 2016;48(5):499–505. 10.1016/j.dld.2016.01.006. 26854256 10.1016/j.dld.2016.01.006

[20] d’Amore F, Relander T, Lauritzsen GF, Jantunen E, Hagberg H, Anderson H, Holte H, Österborg A, Merup M, Brown P, Kuittinen O, Erlanson M, Østenstad B, Fagerli UM, Gadeberg OV, Sundström C, Delabie J, Ralfkiaer E, Vornanen M, Toldbod HE. Up-front autologous stem-cell transplantation in peripheral T-cell lymphoma: NLG-T-01. J Clin Oncol. 2012;30(25):3093–9. 10.1200/JCO.2011.40.2719. 22851556 10.1200/JCO.2011.40.2719

